# Predictors of Trachomatous Trichiasis Surgery Outcome

**DOI:** 10.1016/j.ophtha.2017.03.016

**Published:** 2017-08

**Authors:** Esmael Habtamu, Tariku Wondie, Sintayehu Aweke, Zerihun Tadesse, Mulat Zerihun, Bizuayehu Gashaw, Guadie S. Wondimagegn, Hiwot D. Mengistie, Saul N. Rajak, Kelly Callahan, Helen A. Weiss, Matthew J. Burton

**Affiliations:** 1 London School of Hygiene and Tropical Medicine, London, United Kingdom; 2 The Carter Center, Addis Ababa, Ethiopia; 3 Amhara Regional Health Bureau, Bahirdar, Ethiopia; 4 Bahirdar University, Bahirdar, Ethiopia; 5 Felegehiwot Referral Hospital, Bahirdar, Ethiopia; 6 The Carter Center, Atlanta, Georgia

**Keywords:** BLTR, bilamellar tarsal rotation, CI, confidence interval, ECA, eyelid contour abnormality, FPC, follicles papillae cicatricae, OR, odds ratio, PLTR, posterior lamellar tarsal rotation, PTT, postoperative trachomatous trichiasis, RRR, relative risk ratio, TT, trachomatous trichiasis, WHO, World Health Organization

## Abstract

**Purpose:**

Unfavorable outcomes after trachomatous trichiasis (TT) surgery are undermining the global trachoma elimination effort. This analysis investigates predictors of postoperative TT (PTT), eyelid contour abnormalities (ECAs), and granuloma in the 2 most common TT surgery procedures: posterior lamellar tarsal rotation (PLTR) and bilamellar tarsal rotation (BLTR).

**Design:**

Secondary data analysis from a randomized, controlled, single-masked clinical trial.

**Participants:**

A total of 1000 patients with TT, with lashes touching the eye or evidence of epilation, in association with tarsal conjunctival scarring.

**Methods:**

Participants were randomly allocated and received BLTR (n = 501) or PLTR (n = 499) surgery. Disease severity at baseline, surgical incisions, sutures, and corrections were graded during and immediately after surgery. Participants were examined at 6 and 12 months by assessors masked to allocation.

**Main Outcome Measures:**

Predictors of PTT, ECA, and granuloma.

**Results:**

Data were available for 992 (99.2%) trial participants (496 in each arm). There was strong evidence that performing more peripheral dissection with scissors in PLTR (odd ratio [OR], 0.70; 95% confidence interval [CI], 0.54–0.91; *P* = 0.008) and BLTR (OR, 0.83; 95% CI, 0.72–0.96; *P* = 0.01) independently protected against PTT. Baseline major trichiasis and mixed location lashes and immediate postoperative central undercorrection independently predicted PTT in both surgical procedures. Peripheral lashes in PLTR (OR, 5.91; 95% CI, 1.48–23.5; *P* = 0.01) and external central incision height ≥4 mm in BLTR (OR, 2.89; 95% CI, 1.55–5.41; *P* = 0.001) were independently associated with PTT. Suture interval asymmetry of >2 mm (OR, 3.18; 95% CI, 1.31–7.70; *P* = 0.01) in PLTR and baseline conjunctival scarring in BLTR (OR, 1.72; 95% CI, 1.06–2.81; *P* = 0.03) were independently associated with ECA. Older age was independently associated with ECA in both PLTR (*P* value for trend < 0.0001) and BLTR (*P* value for trend = 0.03). There was substantial intersurgeon variability in ECA rates for both PLTR (range, 19.0%–36.2%) and BLTR (range, 6.1%–28.7%) procedures. In PLTR surgery, irregular posterior lamellar incision at the center of the eyelid (OR, 6.72; 95% CI, 1.55–29.04; *P* = 0.01) and ECA (OR, 3.08; 95% CI, 1.37–6.94; *P* = 0.007) resulted in granuloma formation.

**Conclusions:**

Poor postoperative outcomes in TT surgery were associated with inadequate peripheral dissection, irregular incision, asymmetric suture position and tension, inadequate correction, and lash location. Addressing these will improve TT surgical outcomes.

Visual impairment from trachoma results from the in-turning of eyelashes that scar the cornea (trachomatous trichiasis [TT]). It remains the leading infectious cause of blindness worldwide.^1^, ^2^ Trachomatous trichiasis is a consequence of progressive conjunctival scarring caused by recurrent infection with *Chlamydia trachomatis*. It causes painful corneal abrasion, introduces infection, and alters the ocular surface, eventually leading to irreversible blindness from corneal opacification. Approximately 3.2 million people have untreated TT, and 2.4 million people are visually impaired from trachoma worldwide, of whom an estimated 1.2 million are irreversibly blind.^3^, ^4^

The World Health Organization (WHO) recommends corrective eyelid surgery to reduce the risk of visual impairment from TT.^5^ The surgery involves an incision through the eyelid parallel to and a few millimeters above the lid margin. The terminal portion of the lid is externally rotated and sutured in the corrected position.^6^ In trachoma endemic countries, surgery usually is performed by nonphysician health workers.^6^ There is currently a major global effort to scale up surgical services to clear the current trichiasis backlog by 2020, with more than 200 000 surgeries being performed annually.^7^

However, unfavorable outcomes after TT surgery are undermining these efforts.^8^ Reported rates of postoperative TT (PTT) vary considerably, although 20% recurrence at 1 year is typical.^9^, ^10^, ^11^, ^12^, ^13^, ^14^, ^15^ Moreover, a report from the Global Trachoma Mapping Project suggests the number of cases with unfavorable outcomes, including PTT, have increased significantly with increasing surgical output.^8^ Other adverse outcomes, such as eyelid contour abnormalities (ECAs) and granuloma after surgery, which occurs in 5% to 30% of cases, may have negative social and psychologic impact and deter other patients from accepting trichiasis surgery.^10^, ^13^, ^16^, ^17^, ^18^, ^19^

Several clinical trials have reported unfavorable outcomes to be associated with surgical quality, type of surgical procedure, and preoperative disease severity.^10^, ^11^, ^12^, ^13^, ^14^ The PTT rates between surgeons have been reported to range from 0% to 80%.^10^, ^12^, ^13^, ^14^, ^20^ Management of unfavorable surgical outcomes is often challenging.^8^ First, it requires additional programmatic resources and specialized surgical skills.^8^ Second, the management of postoperative trichiasis is more challenging and probably has poorer outcomes than a primary procedure.^8^ Therefore, every effort must be made to avoid unfavorable outcomes at the primary operation.

We have recently reported the outcome of a randomized controlled trial comparing the bilamellar tarsal rotation (BLTR) and the posterior lamellar tarsal rotation (PLTR) operations.^11^ The PLTR had a substantially lower risk of postoperative trichiasis and was more effective in severe TT cases than BLTR, although BLTR surgery had a lower risk of granuloma formation. The preoperative, intraoperative, and postoperative factors that lead to unfavorable outcomes after TT surgery need to be studied and understood. From the data collected in this trial, we performed a secondary analysis to investigate factors that are associated with unfavorable outcomes (PTT, ECA, and granuloma) after BLTR and PLTR surgery, and identify potential approaches to minimize them.

## Methods

### Ethics Statement

This study was approved by the Ethiopian National Health Research Ethics Review Committee, London School of Hygiene and Tropical Medicine Ethics Committee, and Emory University Institutional Review Board. Written informed consent in Amharic was obtained from participants before enrollment. If a participant was unable to read and write, the information sheet and consent form were read to them and their consent was recorded by thumbprint. An independent Data and Safety Monitoring Committee oversaw the trial. The trial was conducted in compliance with the Declaration of Helsinki and International Conference on Harmonisation–Good Clinical Practice. The trial is registered with the Pan African Clinical Trials Registry (http://www.pactr.org; PACTR201401000743135).

### Study Design and Participants

This was a single-masked, individual-randomized, controlled trial conducted in Ethiopia. The trial methodology has been reported in detail.^11^ Briefly, we recruited 1000 people with TT, with 1 or more lashes touching the eye or evidence of epilation in association with tarsal conjunctival scarring. People with trichiasis due to other causes, recurrent trichiasis after previous surgery, uncontrolled hypertension, pregnancy, and age <18 years were excluded. Recruitment was done through community-based screening in 3 districts of West Gojam Zone, Amhara Region, Ethiopia, between February and May 2014. We trained and standardized 6 trichiasis surgeons in both the PLTR and BLTR surgeries. We used the WHO TT surgery training and certification manual.^6^ Participants were randomized to PLTR or BLTR (using the Waddell clamp) surgery, in a 1:1 allocation ratio for each surgeon. In both surgical procedures, 4/0 silk sutures with 3/8th circle, 19-mm cutting needles were used.

### Clinical Assessments

At baseline, eyes were examined and graded using the Detailed WHO Follicles Papillae Cicatricae (FPC) Grading System.^21^ Lashes touching the eye were counted and subdivided by the part of the eye contacted/location: cornea, lateral, or medial. Trichiasis subtypes were recorded as metaplastic, misdirected, or entropic.^22^ Three trained nurses made intraoperative and immediate postoperative observations. The incision length was measured using a silk suture thread, which was measured against a ruler. The incision height was measured between the incision and the eyelid margin with a sterile ruler. In PLTR surgery, this was measured from the cut edge of the posterior lamella to the lid margin, whereas in BLTR the measurement was done externally on the skin incision. The incision was examined to determine regularity and whether it ran parallel to the lid margin. The scissor cuts made to complete the incision medially and laterally were counted. Data on the number, symmetry, and tension of the sutures were collected. Suture tension was considered “regular” if there was equal tension or firmness across all the sutures and “irregular” if at least 1 of the sutures was insufficiently tight or excessively tight compared with the others. The spacing between sutures was considered “symmetrical” if the difference in space between the central and medial sutures, and the central and lateral sutures was ≤2 mm and “asymmetrical” if this difference was >2 mm. The degree of entropion correction was graded using a previously described system.^11^ Two (primary gaze and up gaze) high-resolution digital photographs of the operated eye were taken before placing the dressing.

Participants were reexamined at 10 days, 6 months, and 12 months postoperatively. At 10 days, PTT, degree of lid eversion, infection, and granulomata were documented before suture removal. At 6 and 12 months, participants were reexamined after the same procedures as for baseline. Eyelid contour abnormalities were graded according to the PRET trial method.^23^ Presenting distance vision was measured at baseline and 12 months using PeekAcuity software on a Smartphone in a dark room.^24^ Three standardized high-resolution digital photographs of trichiasis, cornea, and tarsal conjunctiva were taken, using a Nikon (Tokyo, Japan) D90 digital SLR camera with 105-mm macro lens and R1C1 flash units at baseline and 6- and 12-month follow-ups.^25^

### Statistical Analysis

The sample size was determined on the basis of assumptions described in the primary article of this trial.^11^ Data were double-entered into Access (Microsoft, Redmond, WA) and transferred to Stata 11 (StataCorp LP, College Station, TX) for analysis. For participants who received bilateral surgery, we randomly designated 1 eye to be the study eye for the analysis. The 3 main unfavorable TT surgical outcome measures used in this study are PTT, ECA, and granuloma. Predictors of PTT were defined as 1 or more lashes touching the eye, clinical evidence of epilation, or a history of repeat trichiasis surgery by 12 months. Factors associated with ECA were considered for any type and severity of ECA. In further stratified analysis, moderate and severe ECA were grouped together as “clinically significant” to identify what factors predict these, whereas mild ECA was considered “clinically nonsignificant.” A granuloma was defined as a fleshy tissue growth of at least 2 mm on the tarsal conjunctiva or at the edge of the eyelid. The analysis of ECA was based on the participants seen at 12 months, whereas the analysis of PTT and granuloma included all participants seen at least once during the 6- and 12-month follow-ups.

On the basis of severity, TT cases were categorized into minor trichiasis with <6 lashes or evidence of epilation in less than one-third of the lid margin, and major trichiasis with ≥6 lashes or evidence of epilation in one-third or more of the lid margin. To analyze the association between trichiasis severity and corneal opacity, the detailed corneal opacity grading was converted into the WHO grading system. Mixed postoperative lash location by 12 months were defined as lashes touching more than 1 location at the 6-month or 12-month follow-up or during both follow-ups. The same definition was used for mixed postoperative lash types by 12 months.

Univariable and multivariable association of factors with major trichiasis at baseline, PTT by 12 months, ECA at 12 months, and granuloma by 12 months, and all binary outcomes were analyzed using logistic regression to estimate the odds ratio (OR) and 95% confidence interval (CI) for both surgical procedures separately. Likelihood ratio test was used to decide on the variables that should be included in the final multivariable logistic regression model. *P* value for trend was calculated for ordered categoric exposure variables, such as papillary grade, tarsal conjunctival scar, corneal opacity, visual acuity, and age. Categoric secondary outcomes (e.g., level of correction and ECA severity) were analyzed using multinomial logistic regression to estimate relative risk ratio (RRR) and 95% CI. Correlations between preoperative and postoperative trichiasis lashes location were analyzed using the Fisher exact test because of small observations.

## Results

### Participant Flow

The participant flow for this trial has been described in detail.^11^ In summary, 98% of the participants were examined at all 3 follow-up time points. At 12 months, 491 participants (98.4%) and 490 participants (97.8%) from the PLTR and BLTR arms were reassessed, respectively. The PTT data were available for 992 participants (99.2%), 496 in each arm, who were reassessed on at least 1 occasion during the 12-month period.

### Demographic and Baseline Clinical Characteristics

Baseline demographic and clinical characteristics were analyzed for the 992 trial participants who were seen at 1 or more follow-ups. The majority of the participants were female (758, 76.4%), and the mean age was 47.0 (standard deviation, 14.7) years. Baseline clinical characteristics were balanced between the 2 surgical procedures (Table 1). Major trichiasis was present in 46.4% (230/496) and 48.2% (239/496) of the PLTR and BLTR cases, respectively. Most cases had moderate or severe entropion: PLTR (394/496, 79.4%) and BLTR (406/496, 81.8%). Most individuals in both PLTR (381/496, 76.8%) and BLTR (374/496, 75.4%) groups had lashes in contact with the cornea. Metaplastic lashes were common: PLTR: 223/496 (45.0%), and BLTR: 205/496 (41.3%) (Table 1).

**Table 1:** Clinical and Demographic Characteristics of Cases Seen at Baseline and at 12 Months

Characteristic	Baseline		By 12 Months	
	PLTR	BLTR	PLTR	BLTR
	n/496 (%)	n/496 (%)	n/496 (%)	n/496 (%)
Sex, female	385 (77.6%)	373 (75.2%)	–	–
Age in yrs, mean (SD)	47.0 (15.0)	47.5 (14.9)	–	–
Entropion grade∗				
None/mild (grade 0 and 1)	102 (20.6%)	90 (18.1%)	484 (98·6%)	485 (99.0%)
Moderate (grade 2)	314 (63.3%)	331 (66.7%)	6 (1.2)	5 (1.0%)
Severe (grade 3 and 4)	80 (16.1%)	75 (15.1%)	1 (0.2)	0 (0.0%)
Trichiasis severity				
No trichiasis	–	–	433 (87.3)	386 (77.8%)
Minor trichiasis	266 (53.6%)	257 (51.8%)	56 (11.3)	97 (19.6%)
Major trichiasis	230 (46.4%)	239 (48.2%)	7 (1.41)	13 (2.6%)
Lash location				
No trichiasis	–	–	433 (87.3%)	386 (77.8%)
Epilating	38 (7.7%)	42 (8.5%)	7 (1.4%)	21 (4.2%)
Corneal only	381 (76.8%)	374 (75.4%)	32 (6.5%)	55 (11.1%)
Medial only	3 (0.6%)	0 (0.0%)	12 (2.4%)	7 (1.4%)
Lateral only	8 (1.6%)	5 (1.0%)	5 (1.0%)	5 (1.0%)
Corneal + Peripheral	66 (13.3%)	75 (15.1%)	7 (1.4%)	22 (4.4%)
Lash type				
No trichiasis	–	–	433 (87.3%)	386 (77.8%)
Epilating	38 (7.7%)	42 (8.5%)	7 (1.4%)	21 (4.2%)
Entropic only	126 (25.4%)	117 (23.4%)	5 (1.0%)	8 (1.6%)
Metaplastic only	223 (45.0%)	205 (41.3%)	37 (7.5%)	66 (13.3%)
Misdirected only	9 (1.8%)	13 (2.6%)	2 (0.4%)	4 (0.8%)
Mixed	100 (20.2%)	119 (23.4%)	12 (2.4%)	11 (2.2%)
ECA∗				
None	–	–	371 (75.6%)	404 (82.4%)
Clinically insignificant	–	–	89 (18.1%)	49 (10.0%)
Clinically significant	–	–	31 (6.3%)	37 (7.6%)
Granuloma	–	–	26 (5.2%)	11 (2.2%)

BLTR = bilamellar tarsal rotation; ECA = eyelid contour abnormality; PLTR = posterior lamellar tarsal rotation; SD = standard deviation.

∗ Data analyzed from 12-month examination (PLTR, N = 491; BLTR, N = 490).

Factors associated with preoperative TT severity in all cases are presented in Table S1 (available at www.aaojournal.org). In a multivariable analysis, major TT was significantly associated with being female, increasing corneal opacity, older age, increasing conjunctival scarring, and increasing conjunctival inflammation.

### Postoperative Trachomatous Trichiasis

By 12 months, postoperative trichiasis was present in 173 of 992 cases (17.4%): PLTR 63/496 (12.7%), BLTR 110/496 (22.2%) (Table 1). Most of the cases of PTT by 12 months were minor trichiasis (PLTR: 56/63, 80.9%; BLTR: 97/110, 88.2%). Metaplastic lashes were the most common type of PTT lashes after both procedures: PLTR (37/63, 58.3%) and BLTR (66/110, 60.0%). The most common location of PTT in both PLTR (32/63, 50.8%) and BLTR (55/110, 50.0%) surgery was the cornea (Table 1).

### Posterior Lamellar Tarsal Rotation Surgery

Univariable and multivariable analyses of factors associated with PTT by 12 months after PLTR surgery are presented in Table 2. In a multivariable analysis, there was strong evidence that performing more medial and lateral dissections using scissors to increase the length of surgical incision had a protective effect against PTT (OR, 0.70; 95% CI, 0.54–0.91; *P* = 0.008). The ECA at 12 months was associated with a lower rate of PTT (5.0% vs. 15.4%; OR, 0.24; 95% CI, 0.09–0.60; *P* = 0.002). However, there was evidence that PTT was independently associated with baseline major trichiasis (16.1% vs. 9.8%; OR, 1.97; 95% CI, 1.09–3.56; *P* = 0.03), peripheral lashes (36.4% vs. 10.0%; OR, 5.91; 95% CI, 1.48–23.5; *P* = 0.01), and mixed location lashes (22.8% vs. 10.0%; OR, 2.24; 95% CI, 1.09–4.59; *P* = 0.03) compared with corneal lashes and immediate postoperative central undercorrection compared with adequate correction (36.4% vs. 12.5%; OR, 4.97; 95% CI, 1.15–21.5; *P* = 0.03). Cases with irregular suture tension had a 2 times higher rate of PTT compared with those with regular suture tension (26.7% vs. 12.3%). However, this was not statistically significant (*P* = 0.11). There was no evidence of association between PTT and surgeon (Table 2). Increased severity of baseline conjunctival scarring was significantly associated with major PTT by 12 months (mild, 0/4 [0.0%]; moderate, 4/51 [7.8%]; severe, 3/8 [37.5%]; OR, 7.40; 95% CI, 1.34–40.8; *P* value for trend = 0.02).

**Table 2:** Univariable and Multivariable Association of Factors with Postoperative Trichiasis by 1 Year after Posterior Lamellar Tarsal Rotation Surgery

Demographic and Clinical Factors	PTT N = 496	Univariable Analysis		Multivariable Analysis	
	n/N (%)	OR (95% CI)	*P* Value	OR (95% CI)	*P* Value
Age, yrs					
18–29	3/59 (5.1%)	1.18 (0.98–1.41)	0.08∗	1.23 (1.00–1.50)	0.05∗
30–39	8/85 (9.4%)				
40–49	18/123 (14.6%)				
50–59	17/108 (15.7%)				
60–69	10/74 (13.5%)				
70+	7/47 (14.9%)				
Trichiasis severity					
Minor	26/266 (9.8%)	1.77 (1.04–3.03)	0.04	1.97 (1.09–3.56)	0.03
Major	37/230 (16.1%)				
Lash location					
Epilating	6/38 (15.8%)	1.69 (0.66–4.31)	0.27	1.72 (0.64–4.62)	0.29
Corneal	38/381 (10.0%)	1	–	1	–
Peripheral	4/11 (36.4%)	5.16 (1.44–18.4)	0.01	5.91 (1.48–23.5)	0.01
Corneal + Peripheral	15/66 (22.7%)	2.65 (1.36–5.17)	0.004	2.24 (1.09–4.59)	0.03
Surgeon (relative to surgeon 4)					
1	8/89 (9.0%)	0.81 (0.30–2.16)	0.67	0.57 (0.20–1.63)	0.29
2	14/95 (14.7%)	1.42 (0.60–3.37)	0.43	1.40 (0.56–3.50)	0.48
3	12/84 (14.3%)	1.37 (0.56–3.35)	0.49	0.74 (0.27–2.04)	0.56
4	10/92 (10.9%)	1	–	–	–
5	6/47 (12.8%)	1.20 (0.41–3.53)	0.74	1.62 (0.47–5.55)	0.45
6	13/89 (14.6%)	1.40 (0.58–3.39)	0.45	0.73 (0.27–1.97)	0.54
No. of medial and lateral dissections, median (range)					
No recurrence	1 (0–26)	0.78 (0.63–0.96)	0.02	0.70 (0.54–0.91)	0.008
Recurrence	0 (0–5)				
Immediate postoperative central correction					
Corrected	53/425 (12.5%)	1	–	1	–
Overcorrected	6/60 (10.0%)	0.78 (0.32–1.90)	0.58	0.73 (0.28–1.90)	0.52
Undercorrected	4/11 (36.4%)	4.01 (1.14–14.2)	0.03	4.97 (1.15–21.5)	0.03
Suture tension across sutures					
Regular	59/481 (12.3%)	2.60 (0.80–8.43)	0.11	–	–
Irregular	4/15 (26.7%)				
Surgical incision height in mm∗					
Central					
<4 mm	58/442 (13.1%)	0.66 (0.26–1.77)	0.42	–	–
≥4 mm	5/54 (9.3%)				
ECA					
No	57/371 (15.4%)	0.29 (0.12–0.69)	0.005	0.24 (0.09–0.60)	0.002
Yes	6/120 (5.0%)				

CI = confidence interval; ECA = eyelid contour abnormality; OR = odds ratio; PTT = postoperative trachomatous trichiasis.

Analysis made using logistic regression. The following factors were tested and showed no association with PTT in a univariable analysis (*P* > 0.05) or were excluded from the multivariable analysis after a likelihood ratio test: gender, baseline entropion severity, trichiasis lash type at baseline, baseline conjunctival scarring severity, eye (right, left), number of mattress sutures, and height in millimeters. Age, surgeon, and preoperative trichiasis severity are known to be risk factors for PTT and are included in the multivariable model regardless of significance in a univariable analysis.

∗ *P* value for trend.

There was no evidence of a significant association between surgical incision height of ≥4 mm from the lid margin and PTT, although it tended to give a slightly lower rate of PTT compared with <4 mm incision height (9% vs. 13%) (Table 2). Incision height of <4 mm was not associated with undercorrection. However, a surgical incision height of ≥4 mm was associated with immediate postoperative overcorrection at the corresponding site than a surgical incision height of <4 mm in PLTR surgery: central (27.3% [15/55] vs. 10.1% [45/444]; RRR=3.32; 95% CI, 1.70–6.50; *P* = 0.0004) and lateral (15.6% [7/45] vs. 5.3% [24/454]; RRR, 3.10; 95% CI, 1.25–7.67; *P* = 0.01). Among those with immediate postoperative central overcorrection, 94.9% (56/59) normalized at the 12-month follow-up.

### Bilamellar Tarsal Rotation Surgery

Univariable and multivariable analyses of factors associated with PTT by 12 months in BLTR surgery are presented in Table 3. In a multivariable analysis, there was strong evidence that performing more medial and lateral dissections using scissors to increase the length of surgical incision had a protective effect on PTT (OR, 0.83; 95% CI, 0.72–0.95; *P* = 0.007). Older age (*P* value for trend = 0.007), baseline major trichiasis (31.0% vs. 14.0%; OR, 1.86; 95% CI, 1.12–3.10; *P* = 0.02), mixed location lashes compared with corneal only lashes (46.7% vs. 16.8%; OR, 4.62; 95% CI, 2.51–8.48; *P* < 0.0001), and immediate postoperative central undercorrection compared with adequate correction (60.0% vs. 22.6%; OR, 5.34; 95% CI, 1.32–21.5; *P* = 0.02) were independently associated with PTT (Table 3). Age ≥60 years (OR, 2.05; 95% CI, 1.29–3.25; *P* = 0.002) and major TT at baseline (OR, 2.77; 95% CI, 1.76–4.34; *P* < 0.0001) were independently associated with major PTT by 12 months after BLTR surgery.

**Table 3:** Univariable and Multivariable Association of Factors with Postoperative Trichiasis by 1 Year after Bilamellar Tarsal Rotation Surgery

Demographic and Clinical Factors	PTT N = 496	Univariable Analysis		Multivariable Analysis	
	n/N (%)	OR 95% CI	*P* Value	OR 95% CI	*P* Value
Age, yrs, median (IQR)					
18–29	10/48 (20.8%)	1.26 (1.09–1.46)	0.0005†	1.25 (1.06–1.48)	0.007†
30–39	16/106 (15.1%)				
40–49	17/107 (15.9%)				
50–59	25/103 (24.3%)				
60–69	24/82 (29.3%)				
70+	18/50 (36.0%)				
Trichiasis severity					
Minor	36/257 (14.0%)	2.75 (1.76–4.30)	<0.0001	1.86 (1.12–3.10)	0.02
Major	74/239 (31.0%)				
Lash location					
Epilating	10/42 (23.8%)	1.54 (0.72–3.30)	0.26	1.65 (0.73–3.76)	0.23
Corneal	63/374 (16.8%)	1	–	1	–
Peripheral	2/5 (40.0%)	3.29 (0.54–20.1)	0.20	4.09 (0.60–27.7)	0.15
Corneal + Peripheral	35/75 (46.7%)	4.32 (2.55–7.33)	<0.0001	4.62 (2.51–8.48)	<0.0001
Conjunctival scarring, baseline					
1	8/54 (14.8%)	1.65 (1.09–2.52)	0.02†	1.62 (0.99–2.68)	0.07†
2	78/366 (21.3%)				
3	24/76 (31.6%)				
Surgeon (relative to surgeon 4)					
1	27/91 (29.7%)	1.84 (0.92–3.67)	0.09	1.34 (0.61–2.95)	0.46
2	17/93 (18.3%)	0.97 (0.46–2.05)	0.94	0.86 (0.37–1.99)	0.73
3	17/85 (20.0%)	1.09 (0.51–2.30)	0.82	0.54 (0.22–1.35)	0.19
4	17/91 (18.7%)	1	–	–	–
5	12/47 (25.5%)	1.49 (0.64–3.46)	0.35	2.32 (0.89–6.05)	0.09
6	20/89 (22.5%)	1.26 (0.61–2.61)	0.53	1.22 (0.53–2.81)	0.63
No. of medial and lateral dissections, median (range)†					
No recurrence	2 (0–17)	0.91 (0.82–1.01)	0.06	0.83 (0.72–0.95)	0.007
Recurrence	1 (0–11)				
Immediate postoperative central correction					
Corrected	96/424 (22.6%)	1	–	1	–
Overcorrected	8/62 (12.9%)	0.51 (0.23–1.10)	0.09	0.41 (0.18–0.97)	0.04
Undercorrected	6/10 (60.0%)	5.12 (1.42–18.5)	0.01	5.34 (1.32–21.5)	0.02
Suture tension across sutures					
Regular	105/481 (21.8%)	1.79 (0.60–5.35)	0.30	–	–
Irregular	5/15 (33.3%)				
External surgical incision height in mm∗					
Central					
<4 mm	78/411 (19.0%)	2.58 (1.56–4.26)	0.0002	2.79 (1.52–5.13)	0.001
≥4 mm	32/85 (37.6%)				

CI = confidence interval; IQR = interquartile range; OR = odds ratio; PTT = postoperative trachomatous trichiasis.

Analysis made using logistic regression. The following factors were tested and showed no association with PTT in a univariable analysis (*P* > 0.05) or were excluded from the multivariable analysis after a likelihood ratio test: gender, baseline entropion severity, trichiasis lash type at baseline, eye (right, left), number of mattress sutures, and ECA. Age, surgeon, and preoperative trichiasis severity are known to be risk factors for PTT; therefore, they are included in the multivariable model regardless of significance in a univariable analysis.

∗ Surgical incision height in BLTR surgery was measured externally on the skin.

† *P* value for trend.

In a univariable analysis, lids with external surgical incision height of ≥4 mm from the lid margin were more likely to have PTT compared with those with <4 mm external incision height (central 37.6% vs. 19.0%; medial, 33.3% vs. 19.5%; lateral, 32.1 vs. 19.4%). However, in a multivariable model, this association was only significant for the central external incision height (OR, 2.79; 95% CI, 1.52–5.13; *P* = 0.001) (Table 3). Incision height was not associated with undercorrection or overcorrection in BLTR (data not shown).

### Postoperative Lash Location

**Figure 1:**
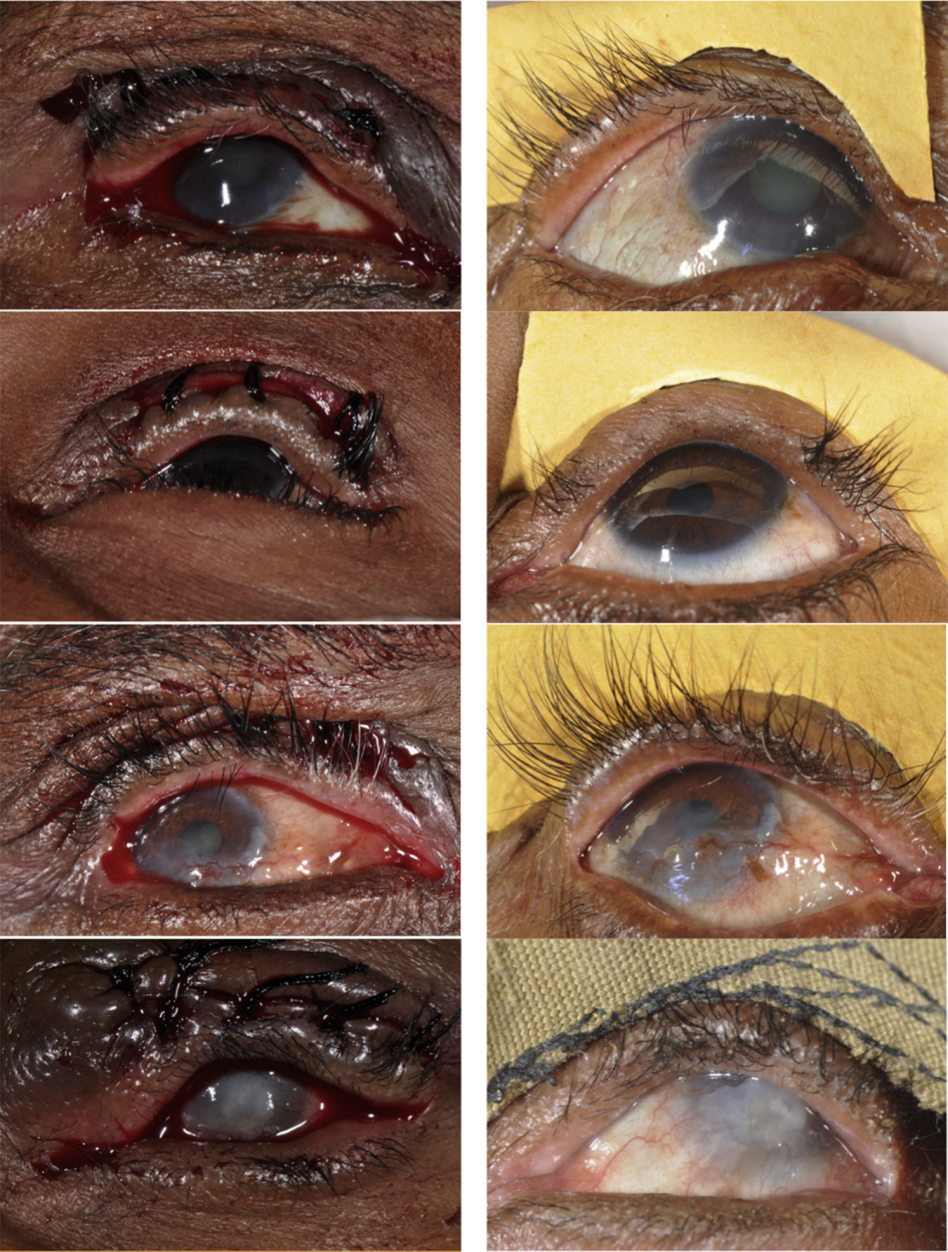
Immediate postoperative undercorrection. **Left**: Immediate postoperative pictures of patient with central undercorrection. **Right**: The 12-month follow-up pictures of the same eye showing postoperative trachomatous trichiasis (PTT) at the same area.

We analyzed whether postoperative trichiasis tended to reoccur in the same sector of the eyelid as it was found preoperatively. Participants with baseline medial only, lateral only, and mixed lashes had a significantly higher rate of postoperative lashes in the same area only compared with those with no baseline lashes in these areas of the eyelid in PLTR: (1) medial (66.7% [2/3] vs. 2.3% [11/493]; Fisher exact test *P* = 0.002); (2) lateral (25.0% [2/8] vs. 0.61% [3/488]; Fisher exact test *P* = 0.002); and (3) mixed location lashes (4.6% [3/66] vs. 0.93% [4/430]; Fisher exact test *P* = 0.05); and in BLTR, mixed location lashes (17.3% [13/75] vs. 2.1% [9/421]; Fisher exact test *P* < 0.001). Figure 1 illustrates this association. However, such correlation was not seen between preoperative and postoperative corneal only lashes for both PLTR (6.8% [26/381] vs. 6.1% [7/115]; Fisher exact test *P* = 1.0) and BLTR (11.0% [41/374] vs. 13.1% [16/122]; Fisher exact test *P* = 0.52) procedures.

The PTT location by 12 months significantly correlates to areas of immediate postoperative undercorrection (Fig 1). Participants with immediate postoperative central undercorrection had a significantly higher rate of central PTT by 12 months than those with immediate postoperative adequate central correction in both PLTR (36.6% [4/11] vs. 7.5% [32/425]; OR, 7.01; 95% CI, 1.95–25.2; *P* = 0.0029) and BLTR (60.0% [6/10] vs. 15.3% [65/424]; OR, 8.28; 95% CI, 2.27–30.2; *P* = 0.0013) surgeries. The medial and lateral analyses were not possible because of insufficient events.

### Eyelid Contour Abnormalities

**Figure 2:**
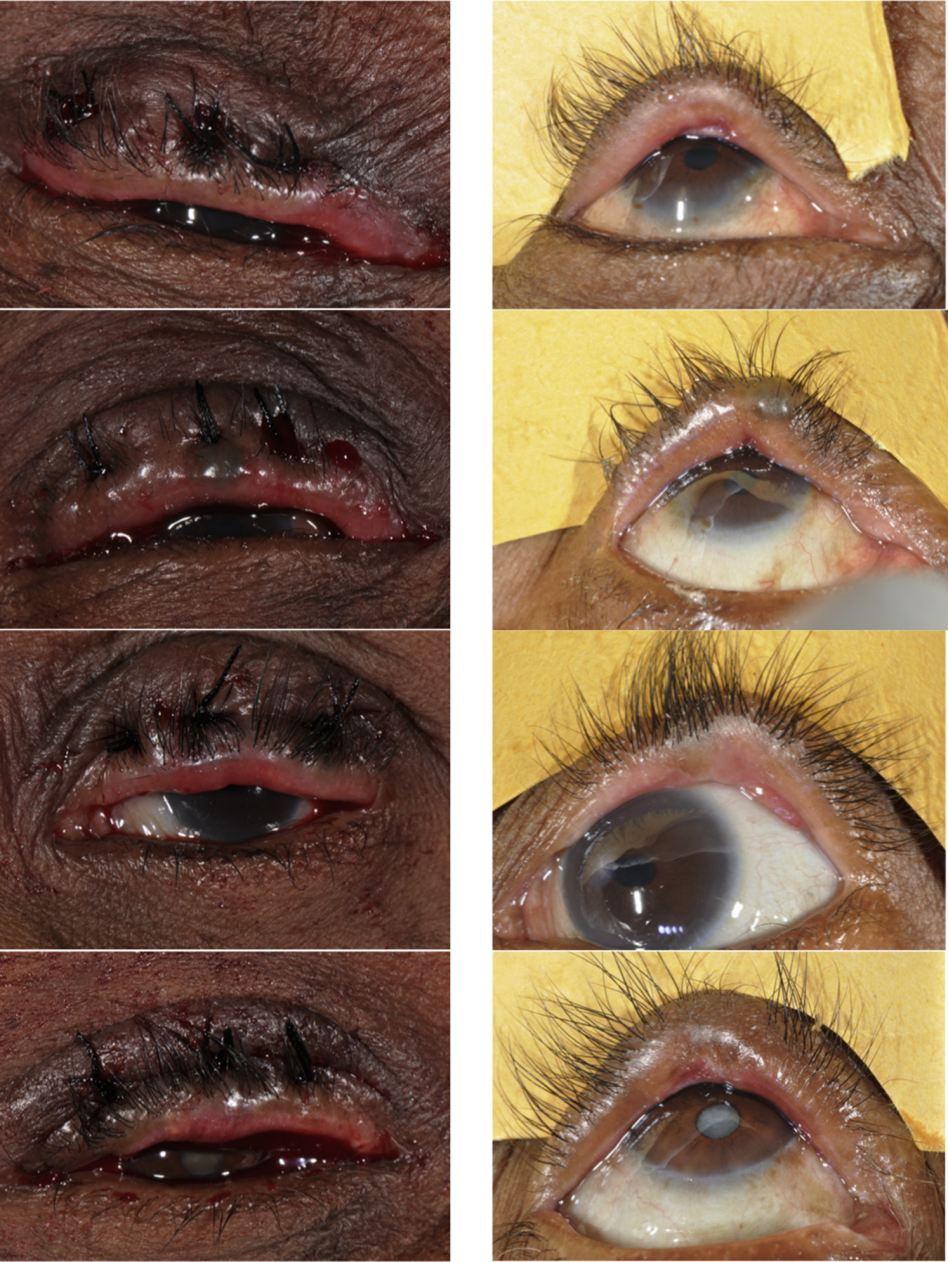
Suture distance asymmetry resulting in eyelid contour abnormality (ECA). **Left**: Immediate postoperative pictures showing asymmetry between suture intervals. **Right**: The 12-month follow-up pictures of the same eye with ECA.

Eyelid contour abnormalities were present in 206 of 981 participants (21.0%) at 12 months: PLTR 120/491 (24.4%) and BLTR 86/490 (17.6%) (Table 1). Univariable and multivariable analyses of factors associated with ECA in PLTR and BLTR surgery are presented in Table 4. Intersurgeon variability is an important factor for ECA in both PLTR (range, 19.0%–36.2%) and BLTR (range, 6.1%–28.7%) procedures (Table 4). In both procedures, old age predicted ECA. Between sutures, distance asymmetry of >2 mm (Fig 2) in PLTR (52.2% vs. 23.1%; OR, 3.18; 95% CI, 1.31–7.70; *P* = 0.003) and baseline conjunctival scarring in BLTR (*P* value for trend = 0.03) were independently associated with ECA. The use of 4 mattress sutures in BLTR surgery halved the rate of ECA compared with 3 mattress sutures (9.5% vs. 19.1%; *P* = 0.03). However, this was not significant in a multivariable analysis (Table 4). In a separate multivariable analysis on the predictors of clinically significant ECA in PLTR surgery, cases aged ≥60 years (12.1% [14/116] vs. 4.5% [17/375]; RRR, 3.91; 95% CI, 1.79–8.56; *P* = 0.0006) and operated by surgeon 5 (17.0% vs. <8%; RRR, 3.23; 95% CI, 1.01–10.4; *P* = 0.049) had a higher risk of developing clinically significant ECA than their counterparts.

**Table 4:** Univariable and Multivariable Association of Factors with Eyelid Contour Abnormality at 1 Year by Type of Surgery

Demographic and Clinical Factors	PLTR (N = 491)					BLTR (N = 490)				
	ECA	Univariable		Multivariable		ECA	Univariable		Multivariable	
	n/N (%)	OR 95% CI	*P* Value	OR 95% CI	*P* Value	n/N (%)	OR 95% CI	*P* Value	OR 95% CI	*P* Value
Age (yrs), continuous										
18–29	8/59 (13.6%)	1.39 (1.20–1.62)	<0.0001†	1.39 (1.20–1.62)	<0.0001†	5/48 (10.4%)	1.14 (0.98–1.34)	0.09†	1.20 (1.02–1.42)	0.03†
30–39	11/85 (12.9%)					17/106 (16.0%)				
40–49	27/123 (21.9%)					18/106 (17.0%)				
50–59	32/108 (29.6%)					20/101 (19.8%)				
60–69	25/73 (34.2%)					14/81 (17.3%)				
70+	17/43 (39.5%)					12/48 (25.0%)				
Conjunctival scarring, baseline										
1	13/47 (27.7%)	0.74 (0.48–1.13)	0.16†	–	–	5/52 (9.6%)	1.52 (0.96–2.42)	0.08†	1.72 (1.06–2.81)	0.03†
2	94/370 (25.4%)					65/366 (17.7%)				
3	13/74 (17.6%)					16/72 (22.2%)				
Surgeon (relative to surgeon 3)										
1	21/86 (24.4%)	1.37 (0.66–2.86)	0.40	1.13 (0.53–2.44)	0.74	10/91 (11.0%)	1.90 (0.62–5.81)	0.26	1.87 (0.60–5.81)	0.28
2	27/95 (28.4%)	1.69 (0.83–3.41)	0.14	1.60 (0.78–3.28)	0.20	25/92 (27.2%)	5.75 (2.08–15.9)	0.0007	5.84 (2.07–16.4)	0.0008
3	16/84 (19.0%)	–	–	–	–	5/82 (6.1%)	1	–	–	–
4	19/90 (21.1%)	1.14 (0.54–2.39)	0.73	0.99 (0.46–2.14)	0.98	11/91 (12.1%)	2.12 (0.70–6.38)	0.18	2.13 (0.70–6.52)	0.18
5	17/47 (36.2%)	2.41 (1.08–5.40)	0.03	2.39 (1.04–5.49)	0.04	10/47 (21.3%)	4.16 (1.33–13.05)	0.014	4.40 (1.37–14.2)	0.01
6	20/89 (22.5%)	1.23 (0.59–2.58)	0.58	1.15 (0.54–2.44)	0.72	25/87 (28.7%)	6.21 (2.25–17.2)	0.0004	5.81 (2.07–16.3)	0.0009
No. of mattress sutures										
3 sutures	103/396 (26.0%)	0.65 (0.37–1.16)	0.15	–	–	74/388 (19.1%)	0.44 (0.21–0.92)	0.030	–	–
4 sutures	17/91 (18.7%)					9/95 (9.5%)				
Suture distance										
Symmetric	108/468 (23.1%)	3.64 (1.56–8.47)	0.0028	3.18 (1.31–7.70)	0.01	77/457 (16.8%)	1.85 (0.83–4.14)	0.13	–	–
Asymmetric∗	12/23 (52.2%)					9/33 (27.3%)				

BLTR = bilamellar tarsal rotation; CI = confidence interval; ECA = eyelid contour abnormality; OR = odds ratio; PLTR = posterior lamellar tarsal rotation.

Analysis made using logistic regression. The following factors were tested and showed no association with ECA in a univariable analysis (*P* > 0.05) or were excluded from the multivariable analysis after a likelihood ratio test: gender, baseline trichiasis severity, baseline conjunctival scarring severity, suture tension across sutures, and surgical incision height in millimeters. Surgeon and age were included in the multivariable analysis regardless of significance in univariable analysis because these are known to be associated with ECA from previous studies.

Dashed lines indicated that variables are excluded from the final model after likelihood ratio test.

∗ Space between >2 mm symmetry difference between each other.

† *P* value for trend.

### Granuloma

Granuloma was documented in 37 of 992 participants (3.7%) during the 12-month period: PLTR 26/496 (5.2%) and BLTR 11/496 (2.2%) (Table 1). The development of a granuloma after PLTR surgery was independently associated with (1) a posterior lamellar incision that was irregular or not parallel to the lid margin in the central one-third of the eyelid (OR, 6.72; 95% CI, 1.55–29.04; *P* = 0.01) and (2) ECA (OR, 3.08; 95% CI, 1.37–6.94; *P* = 0.007) (Table 5). There was a nonsignificant trend for cases with suture tension irregularity having a higher rate (3-fold) of granuloma compared with those with regular suture tension in both PLTR (5.0% vs. 13.3%) and BLTR (2.1% vs. 6.7%) procedures. Preoperative disease severity, age, and surgeon were not associated with granuloma in both surgeries. There were no significant associations with granuloma after BLTR (Table 5).

**Table 5:** Univariable and Multivariable Association of Factors with Granuloma in 1 Year, by Type of Surgery

Demographic and Clinical Factors	PLTR (N = 496)					BLTR (N = 496)		
	Granuloma	Univariable		Multivariable		Granuloma	Univariable	
	n/N (%)	OR 95% CI	*P* Value	OR 95% CI	*P* Value	n/N (%)	OR 95% CI	*P* Value
Age, yrs, continuous								
18–29	4/59 (6.8%)	1.00 (0.76–1.30)	0.98∗	–	–	0/48 (0.0%)	1.20 (0.79–1.79)	0.39∗
30–39	5/85 (5.9%)					1/106 (0.9%)		
40–49	5/123 (4.1%)					3/107 (2.8%)		
50–59	3/108 (2.8%)					4/103 (3.9%)		
60–69	7/74 (9.5%)					3/82 (3.7%)		
70+	2/47 (4.3%)					0/50 (0.0%)		
Surgeon (relative to surgeon 3)								
1	5/89 (5.6%)	1.82 (0.42–7.87)	0.42	–	–	2/91 (2.2%)	1.90 (0.62–5.81)	0.26
2	3/95 (3.2%)	1	–	–	–	1/93 (1.1%)	1	–
3	9/84 (10.7%)	3.68 (0.96–14.1)	0.42	–	–	3/85 (3.5%)	3.37 (0.34–33.0)	0.30
4	3/92 (3.3%)	1.03 (0.20–5.26)	0.97	–	–	2/91 (2.2%)	2.07 (0.18–23.2)	0.56
5	2/47 (4.3%)	1.36 (0.22–8.45)	0.74	–	–	1/47 (2.1%)	2.00 (0.12–32.7)	0.63
6	4/89 (4.5%)	1.44 (0.31–6.34)	0.64	–	–	2/89 (2.2%)	2.11 (0.19–23.7)	0.54
Central incision in relation to lid margin								
Regular/parallel	23/486 (4.7%)	8.6 (2.1–35.5)	0.003	6.72 (1.55–29.0)	0.01	1/13 (7.7%)	–	–
Irregular/unparalleled or slanted	3/10 (30.0%)					25/483 (5.2%)		
Suture tension across sutures								
Regular	24/481 (5.0%)	2.93 (0.62–13.7)	0.17	–	–	10/481 (2.1%)	3.36 (0.40–28.1)	0.26
Irregular	2/15 (13.3%)					1/15 (6.7%)		
ECA								
No	13/371 (3.5%)	3.35 (1.51–7.43)	0.003	3.08 (1.37–6.94)	0.007	8/404 (2.0%)	1.79 (.46–6.89)	0.40
Yes	13/120 (10.8%)					3/86 (3.5%)		

BLTR = bilamellar tarsal rotation; CI = confidence interval; ECA = eyelid contour abnormality; OR = odds ratio; PLTR = posterior lamellar tarsal rotation.

Analysis made using logistic regression. The following factors were tested and showed no association with granuloma in a univariable analysis (*P* > 0.05) or were excluded from the multivariable analysis after a likelihood ratio test: gender, baseline trichiasis severity, suture distance asymmetry, and surgical incision height in millimeters. Multivariable analysis for BLTR surgery is not presented because none of the tested factors showed significant association in univariable analysis.

Dashed lines indicated that variables are excluded from the final model after likelihood ratio test.

∗ *P* value for trend.

## Discussion

Poor outcomes from TT surgery affect both the individual and the trachoma control program as a whole. A good understanding of the factors that increase the likelihood of an adverse outcome is crucial for surgeons, surgical trainers, and program planners. In this study, we explored factors associated with postoperative trichiasis, ECA, and granuloma formation after PLTR and BLTR surgeries. The results showed a range of intraoperative and immediate postoperative factors are probably vital in shaping trichiasis surgery outcomes.

### Postoperative Trachomatous Trichiasis

#### Peripheral Dissections

Perhaps one of the most useful findings of this study was that extending the length of the incision medially and laterally markedly reduced the rate of PTT for both PLTR and BLTR. A longer incision allows the distal segment to rotate adequately, and once secured with sutures, it is less likely to revert to the original entropic position. In addition, the longer incision probably allows the most medial and lateral extents of the lid to rotate more freely, thereby successfully correcting peripheral trichiasis.

Baseline lash location was an important determinant of PTT for both procedures. Peripheral TT lashes and mixed location lashes were substantially more likely to recur than corneal only lashes in PLTR surgery, whereas mixed location lashes were more likely to recur than corneal only lashes in BLTR surgery. This may be due to an insufficiently long surgical incision, failing to correct the trichiasis at the peripheries of the eyelid. An earlier study has reported comparable results, in which eyelids with shorter incisions had a 4-fold higher rate of recurrent TT than those with longer incision length.^26^ Surgical training must emphasize the importance of the peripheral incision and dissection achieving adequate rotation.

#### Incision Height

There was a trend toward lower recurrence rate in cases with an incision height of ≥4 mm from the lid margin in PLTR surgery. A higher incision height creates a larger distal segment that rotates more freely, pulling the lashes further away from the globe. In contrast, BLTR surgery cases with a central incision height of ≥4 mm had approximately 2 times more PTT than those with <4-mm incision height. The differential result of incision height between PLTR and BLTR surgery concurs with a previous prospective cohort study that reported a 72% (275/380) success rate in patients with mild to moderate trachomatous cicatricial entropion operated using BLTR with an incision made at >3 mm distance from the lid margin (on the eyelid crease) and an 82% (410/500) success rate in patients with moderate to severe cicatricial entropion using PLTR with an incision made at approximately 2 mm from the grey line.^27^ There are several possible explanations for this seemingly paradoxical finding. First, it is possible that irrespective of incision height, the distal fragment rotates less freely in BLTR than PLTR perhaps because the procedure does not incorporate dissection between the 2 lamellae. Without this dissection, there may still be too much tension holding the large distal segment or pulling it back to the entropic position. Second, in our study, the incision height in BLTR was measured from the skin immediately after the incision. However, this distance may not be accurate because of the distension of the skin by the local anesthetic; therefore, this might give a different measure of the incision height than the measure in the posterior lamella because the posterior lamella anatomy is not distorted by the local anesthetic injection. Third, higher incisions in BLTR may result in overcorrection. Therefore, the surgeons may deliberately under-tighten the everting sutures as per the WHO training manual, resulting in undercorrection as the postoperative swelling settles.^6^ Overall, these results indicate that the current WHO-recommended surgical incision height of approximately 3 mm should be maintained for both PLTR and BLTR surgeries. For BLTR surgery, the height of the incision to be made should be marked before the infiltration of anesthesia.

#### Suture Tension

Irregular suture tension at the end of surgery was associated with a trend toward more PTT in both PLTR and BLTR surgeries. Suture tension is an essential part of entropion correction in TT surgery. Irregularly and loosely tied sutures will become apparent as the swelling settles and will fail to hold the distal portion of the eyelid in the desired position, resulting in undercorrection or PTT. There are no previous reports on the effect of suture tension on surgical outcomes. Our study supports the WHO trichiasis surgery training manual recommendation that all sutures should be “tightened firmly enough to produce a slight overcorrection,” although we would consider omitting the word “slight” particularly for more severe cases and adding the importance of the sutures being evenly spaced (discussed later).

#### Patient-Related Factors

Preoperative major trichiasis and older age were independent predictors of PTT for both surgical procedures in this and other clinical trials.^10^, ^12^, ^14^, ^17^, ^26^, ^28^, ^29^, ^30^ These were also predictors for major PTT in BLTR (but not in PLTR) surgery in this study, suggesting that such cases probably should be treated with PLTR. This is consistent with the literature that cases with severe disease should be treated with surgical procedures concentrating on the posterior lamella to minimize failure because it would effectively correct severe entropion with 180^°^ rotation to the distal tarso-conjunctiva.^27^, ^31^, ^32^, ^33^ However, BLTR was considered effective for relatively less severe cases.^11^, ^27^, ^31^, ^32^, ^33^ Operating on severe trichiasis cases is more challenging because of severe tarsal conjunctival scarring and associated lid shortening.^26^, ^28^ Severe conjunctival scarring and old age may affect wound healing, resulting in the eyelid reverting to its entropic position.^26^, ^28^ Preoperative major trichiasis and older patients ideally should be treated by the most experienced surgeon available to minimize failure.

### Eyelid Contour Abnormality

An ECA is an undesirable outcome of TT surgery even if the TT has been corrected. It is cosmetically unsightly and probably uncomfortable because it will affect tear film distribution and deter others from undergoing TT surgery.^16^ As the sutures are tightened in PLTR surgery, the larger superior posterior lamella portion is pulled toward and tucked under the smaller distal portion, which both assists with rotating the distal portion and prevents the sutures being in contact with the cornea. Unequal suture positioning probably creates unbalanced forces that tucks the proximal fragment to a variable degree, resulting in lid margin distortion.^28^ Marking where the suture should be positioned before injecting local anesthesia should be practiced to avoid suture asymmetry. The use of 4 mattress sutures tended to reduce ECA, probably because there is less opportunity for uneven suture placement. Further studies are needed to establish the effect of 4 mattress sutures in TT surgery. The ECA rates for both PLTR and BLTR varied by surgeons. In our study, 2 surgeons had consistently higher rates of ECA for both procedures. This indicates that despite rigorous training and extensive experience, subtle differences in how the incisions are made and the sutures are positioned and tightened can result in ECA. In addition, the primary objective of TT surgery is to treat the TT. Therefore, trainings and standardizations often focus more on how to achieve effective outward rotation to avoid surgical failure and recurrence than on prevention of ECA. Eyelid contour abnormalities were more frequent in older people after PLTR and BLTR surgery in this study, possibly because older individuals already have more distorted eyelids from the more advanced tarsal scarring or because the older eyelid is more pliable and therefore more susceptible to irregular suture tensions and forces. Similar results have been reported in earlier studies that used BLTR.^26^, ^28^

### Granuloma

Granuloma is more common in PLTR. This is consistent with findings in other studies.^19^, ^27^, ^28^ In this study, there was evidence that an irregular/slanted posterior lamellar incision in the central third of the eyelid increased the rate of granuloma formation by more than 6-fold in PLTR surgery. These cases probably have a larger posterior lamella defect that supports the mechanism of granuloma formation after PLTR surgery than we had previously hypothesized, with granulomas developing from the subepithelial tissue where there is a persistent defect, and perhaps representing over-exuberant healing responses to fill the defects.^11^ It also has been reported that a gap between the incised edge of the posterior lamella from inadequate suturing in PLTR surgery may lead to granuloma formation.^34^ Granuloma was less likely after BLTR, presumably because the operation, which used the Waddell clamp, has less conjunctival manipulation and does not leave a significant conjunctival defect, unless there is irregular suture tension, in which case the risk of granuloma is higher.^13^

### Study Strengths and Limitations

This study has a high follow-up rate of large cohort of patients, providing good power to examine the determinants of the outcomes of both PLTR and BLTR surgery. The potential design limitations of such a surgical trial, such as the risk of unmasking during the operation, and in the follow-ups due to surgical scars have been discussed in detail.^11^ However, independent photograph grading analysis showed there was no evidence of systematic bias in the field grading.^11^ Moreover, procedure unmasking is less likely to be an issue in this particular analysis because determinants of surgical outcome were analyzed within each surgical procedure. Another limitation is that the surgical incision in BLTR surgery was measured externally on the skin after the injection of the local anesthesia, which could be a more variable measure than the incision and scar height on the posterior lamella. The suture tension grading probably is less objective than other grading measures used in this study. It is possible that other unstudied factors in this trial, such as genetic predisposition, imbalance in the initial wound-healing process, and progression of conjunctival inflammation and scarring, could influence TT surgical outcomes. We cannot rule out the possibility that some of the associations occurred by chance because we evaluated multiple risk factors.

There is currently an unprecedented global effort to improve TT surgical quality and reduce the number of people developing PTT who would rejoin the TT backlog. The findings of this study contribute to trachoma-control programs by helping to identify modifiable operative factors that influence outcome. Improving outcomes probably will promote uptake and reduces the overall cost to the patients and program. The major surgical factors that are found to affect outcomes in this study are relatively straightforward to address during surgical training and surgical practice and should be incorporated into TT surgery programs. These should be included in the WHO surgeon standardization checklist as mandatory items for certification. Surgical outcome monitoring should be strengthened. A system for frequent and regular supportive supervision and active follow-up of patients should be implemented. Surgeons with consistently poor surgical outcomes should be identified and given additional practical training. Further research is needed on how to improve surgical outcomes, on the effect of immediate postoperative correction of an unfavorable outcome, and on how to manage PTT and clinically significant ECA.
