# Supplementary material for: Predictors of Trachomatous Trichiasis Surgery Outcome
Source: Ophthalmology. 2017 Aug;124(8):1143–55. doi: 10.1016/j.ophtha.2017.03.016 (PMC5540045; doi:10.1016/j.ophtha.2017.03.016)
Supplement: Table S1 [file mmc1.pdf]

Supplemental Table S1: Univariable and multivariable analysis of baseline factors associated with preoperative TT severity

Variable	Minor TT		Major TT		Univariable Analysis			Multivariable analysis		
	n/525	*(%)*	*n/475*	*(%)*	*OR*	*(95% CI)*	*P-value*	*OR*	*95% CI*	*P-value*
Sex, Female	372	(70.9%)	393	(82.7%)	1.97	(1.45 – 2.67)	<0.0001	2.29	(1.64 – 3.21)	<0.0001
Age, mean (SD)	46.1	(14.6%)	48.7	(15.2%)	1.01	(1.01 – 1.03)	0.005	1.02	(1.01 – 1.03)	0.02
Lash location										
Corneal only	428	(81.5%)	331	(69.7%)	0.52	(0.39 – 0.70)	<0.0001	0.65	(0.41 – 1.05)	0.08
Peripheral only	10	(1.9%)	6	(1.3%)	0.66	(0.24 – 1.83)	0.42	-	-	-
Mixed	46	(8.8%)	97	(20.4%)	2.67	(1.83 – 3.89)	<0.0001	-	-	-
Lash type										
Entropic	146	(27.8%)	97	(20.4%)	0.67	(0.50 – 0.89)	0.007	0.70	(0.49 – 1.01)	0.06
Metaplastic	229	(43.6%)	201	(42.3%)	0.95	(0.74 – 1.22)	0.68	-	-	-
Misdirected	21	(4.0%)	2	(0.4%)	0.10	(0.02 – 0.43)	0.002	0.22	(0.05 – 0.99)	0.05
Mixed	88	(16.7%)	134	(28.2%)	1.95	(1.44 – 2.64)	<0.0001	1.45	(1.01 – 2.06)	0.05
Lower lid TT	36	(6.9%)	54	(11.4%)	1.74	(1.12 – 2.71)	0.01	1.52	(0.94 – 2.44)	0.09
Papillary grade										
None	10	(1.9%)	5	(1.1%)	1.84	(1.50 – 2.25)	<0.0001	1.54	(1.23 – 1.94)	0.0002†
Mild	167	(31.8%)	81	(17.1%)						
Moderate	298	(56.8%)	305	(64.2%)						
Severe	50	(9.5%)	84	(17.7%)						
Tarsal conjunctiva scar										
Mild	76	(14.5%)	31	(6.5%)	2.23	(1.72 – 2.91)	<0.0001	2.08	(1.54 – 2.82)	<0.0001†
Moderate	398	(75.8%)	342	(72.0%)						
Severe	51	(9.7%)	102	(21.5%)						
Corneal scar										
CCO	171	(32.6%)	82	(17.3%)	1.61	(1.37 – 1.89)	<0.0001	1.30	(1.09 – 1.56)	0.004†
CC1	205	(39.1%)	200	(42.1%)						
CC2	143	(27.2%)	173	(36.4%)						
CC3	6	(1.1%)	20	(4.2%)						
Best corrected logMAR visual acuity										
-1.0 – 0.29	169	(32.2%)	109	(22.9%)	1.19	(1.07 – 1.33)	0.002	-	-	-
0.3 – 0.69	207	(39.4%)	192	(40.4%)						
0.7 – 1.0	94	(17.9%)	116	(24.4%)						
1.1 – 1.9	22	(4.2%)	14	(2.9%)						
2.0 – 3.0/CF, HM, LP	29	(5.5%)	35	(7.4%)						
3.5/NLP	4	(0.8%)	9	(1.9%)						

Note: Analysis made using logistic regression. † P- value for trend
